# Football training vs. martial arts training: How are they related to executive function skills in 5–6-year-old boys?

**DOI:** 10.3389/fpsyg.2025.1668266

**Published:** 2025-10-15

**Authors:** Anastasia Yakushina, Natalia Rudnova, Maria Dmitrieva, Aleksander Pashenko, Elena Chichinina

**Affiliations:** ^1^Federal Scientific Center of Psychological and Multidisciplinary Research (FSC PMR), Moscow, Russia; ^2^Faculty of Psychology, Lomonosov Moscow State University, Moscow, Russia

**Keywords:** executive function skills, open-skilled sports, football training, martial arts training, boys

## Abstract

Sports training is one of the most popular extracurricular activities among preschool and elementary school children. The aim of this pilot study was to compare executive function skills in preschool boys who participated in football training and those who undertook martial arts training. The participants were sixty (60) typically developing 5–6-year-old boys from large Russian cities. There were two groups with 30 boys in each group: a football group and a martial arts group. Inclusion criteria were as follows: (a) male gender; (b) boys did not attend any extracurricular activities other than their football training or martial arts training; (c) the duration of football or martial arts training session was more than 30 min; (d) boys attended these trainings for at least 6 months and no more than 2 years. Caregivers reported on the specifics of child’s attendance at sports training and child’s age, maternal education, and family income, which were used as control variables. The results indicated that boys involved in football training had significantly higher spatial component of visuospatial working memory compared to boys involved in martial arts training. Taken together, these findings suggest that football training compared to martial arts training in 5–6-year-old boys may have more potential in terms of developing spatial component of visuospatial working memory. The results of this pilot study could serve as a basis for further investigation on this topic.

## 1 Introduction

Extracurricular activities are an important source of children’s development outside of kindergarten (Allen et al., 2022). Extracurricular activities refer to structured and regular trainings with a schedule in clubs and sections under the guidance of a suitable adult (Polivanova et al., 2016; Weininger et al., 2015). Extracurricular activities include sports, music, dance, art, language learning and other (Weininger et al., 2015). According to data from the US Census Bureau, in the USA, 57% of children aged 6 to 17 were involved in one or more extracurricular activities. Among these participants, 35% engaged in sports, 29% took lessons such as music, dance, or language, another 29% joined clubs, such as religious youth groups and Scouts (A Child's Day: Historical Tables and Figures, 2022). In Europe, around 95% of preschoolers take part in some form of early childhood education, and also engage in extracurricular activities such as sports, music, arts, and other types of enrichment programmes (Early childhood education statistics, 2025). Sports training is one of the most popular extracurricular activities among children throughout the world (Felfe et al., 2016). Moreover, the number of children between 3 and 12 years old who systematically participate in sports is increasing every year (Sabo and Veliz, 2008). This is a positive trend, as sports training can have a positive impact on both the physical and mental development of children (Liersch et al., 2025; Yakushina et al., 2024; Ríos-Garit et al., 2024).

The positive effects of sports training on mental development can be explained by several reasons. Firstly, physical activity induces functional changes in the brain which positively affects neuroplasticity, leading to improved cognitive performance (Chaddock-Heyman et al., 2016; Vasilopoulos et al., 2023; Tan et al., 2025). Secondly, sports training is a structured activity that requires interaction with others, self-control, and cognitive load (Diamond and Ling, 2016). Thus, sports training may have great potential for executive function skills (EF skills) development, which are crucial for successful school adjustment and determine academic and social achievements (Kim and Tsethlikai, 2025; Sasser et al., 2015).

EF skills are one of the most important developmental indicators in early childhood (Panfilova et al., 2024; Sadozai et al., 2024; Zelazo et al., 2017). EF skills are a group of cognitive abilities that ensure purposeful behavior, helping to predict future academic and social achievements (Oshchepkova and Shatskaya, 2023; Robson et al., 2020; Diamond, 2013). According to Miyake’s model, the following EF skills are distinguished: inhibition, working memory, and cognitive flexibility (Miyake et al., 2000). Preschool age is the most sensitive age for the development of EF skills (Klopotova and Yaglovskaya, 2024; Bukhalenkova et al., 2023). Therefore, their development in preschool and elementary school years through sports training is promising (Contreras-Osorio et al., 2021). A large number of studies confirm that sports activities contribute to the development of EF skills (Hu et al., 2025; Giordano et al., 2021). However, it has not been established which sports trainings are most effective in developing EF skills.

Improving EF skills through sports training depends on which skills are involved in sport (Rahimi et al., 2022). Depending on the extent to which external conditions influence the execution of movements, sports can be divided into open-skilled and closed-skilled (Hu et al., 2025). Open-skilled sports (e.g., game sports, martial arts) involve rapid and constant change of actions depending on the opponent’s actions and the environment (Lai et al., 2024). In contrast, closed-skilled sports (running, cycling, swimming) require less cognitive input and are performed in a much more predictable environment (Heilmann et al., 2022). Recent studies show that engaging in open-skilled sports may enhance EF skills (Hu et al., 2025; Lai et al., 2024; Mohring et al., 2022). So, open-skilled sports require quick decision-making, anticipation, planning and adapting to rapidly changing conditions, which particularly favors the development of EF skills (Lai et al., 2024; Heilmann et al., 2022; De Waelle et al., 2021).

Despite the research findings mentioned earlier, little is known about the impact of specific open-skilled sports on children’s EF skills. In particular, a number of studies have suggested positive effects of team sports such as football, basketball, volleyball and ice hockey (Wu et al., 2025; Glavaš et al., 2023; De Waelle et al., 2021). Other studies, however, emphasize the benefits of martial arts training (Pinto-Escalona et al., 2024; Giordano et al., 2021). This is because martial arts develop not only physical attributes but are also associated with the development of will and self-control (Ortiz-Franco et al., 2024; Diamond, 2012). Based on the review of studies, it remains unclear what benefits various open-skilled sports have for the development of EF skills in preschoolers.

## 2 Current study

Studying which sports training is most favorable for the development of preschoolers’ and elementary school children’s EF skills opens up the prospect of successful adaptation to school. Therefore, the purpose of this pilot study was to compare the EF skills in 5–6-year-old children who participated in football training and martial arts training. Martial arts and football are some of the most popular open-skilled sports trainings among 5–6-year-old children (Felfe et al., 2016). The 2025 survey results indicated that in Russia 23% of respondents’ children participated in martial arts, while 19% were involved in football (Forbes Sport, 2024). At the same time, these sports are predominantly practiced by boys, which is why only boys were included in the sample. The effect of sports training on EF skills depends not only on the type of sport, but also on the duration and period of training attendance (Diamond and Ling, 2016). Therefore, we included boys who have been attending sports training for at least six months in the study. The specifics of attendance at sports training, such as number of sessions per week, the duration of one session in minutes, and the time the child has been attending training in months, were controlled in the study. Potential individual and family characteristics that could also influence EF skills development in 5–6-year-old children, including maternal education, child’s age, and family income, were also controlled in the study. In order to achieve the aim of the pilot study, we posed the following research question: How do EF skills differ between 5 and 6-year-old boys attending football training and boys attending martial arts training?

## 3 Materials and methods

### 3.1 Procedure

The study was conducted in seven kindergartens in three large Russian cities (Kazan, Moscow, Sochi). Caregivers of all children in the kindergartens available for the study were sent the questionnaire by e-mail. This questionnaire contained questions about the children’s attendance of extracurricular activities. The questionnaire also included further questions about the main socio-demographic characteristics of the family and the child.

Based on the responses of the caregivers to the questions, the sample for the study was prepared from normally developing children who met the inclusion criteria and who were also fluent in Russian. Inclusion criteria for the groups were as follows: (a) male gender; (b) boys did not attend any extracurricular activities other than football training or martial arts training; (c) the duration of football or martial arts training session was more than 30 min; (d) boys attended these trainings for at least 6 months and no more than 2 years. The upper age limit of 2 years was due to the fact that only boys who were involved in football training had a period of attendance of more than 2 years.

Children who met the inclusion criteria took part in the EF skills assessment. The assessment was conducted by pre-trained psychologists. The sequence of tests was always the same: visuospatial working memory assessment, verbal working memory assessment, inhibition assessment, and cognitive flexibility assessment. The EF skills assessment was conducted in the morning hours in the kindergarten, in a familiar quiet place with good lighting, such as in a psychologist’s office or quiet room.

Before the EF skills assessment, all caregivers gave their written informed consent for their child’s participation in the study. This study and its consent procedures were approved by the Ethics Committee of Federal Scientific Center of Psychological and Interdisciplinary Research (approval No: 3 dated 10 January 2025).

### 3.2 Participants

**Figure 1:**
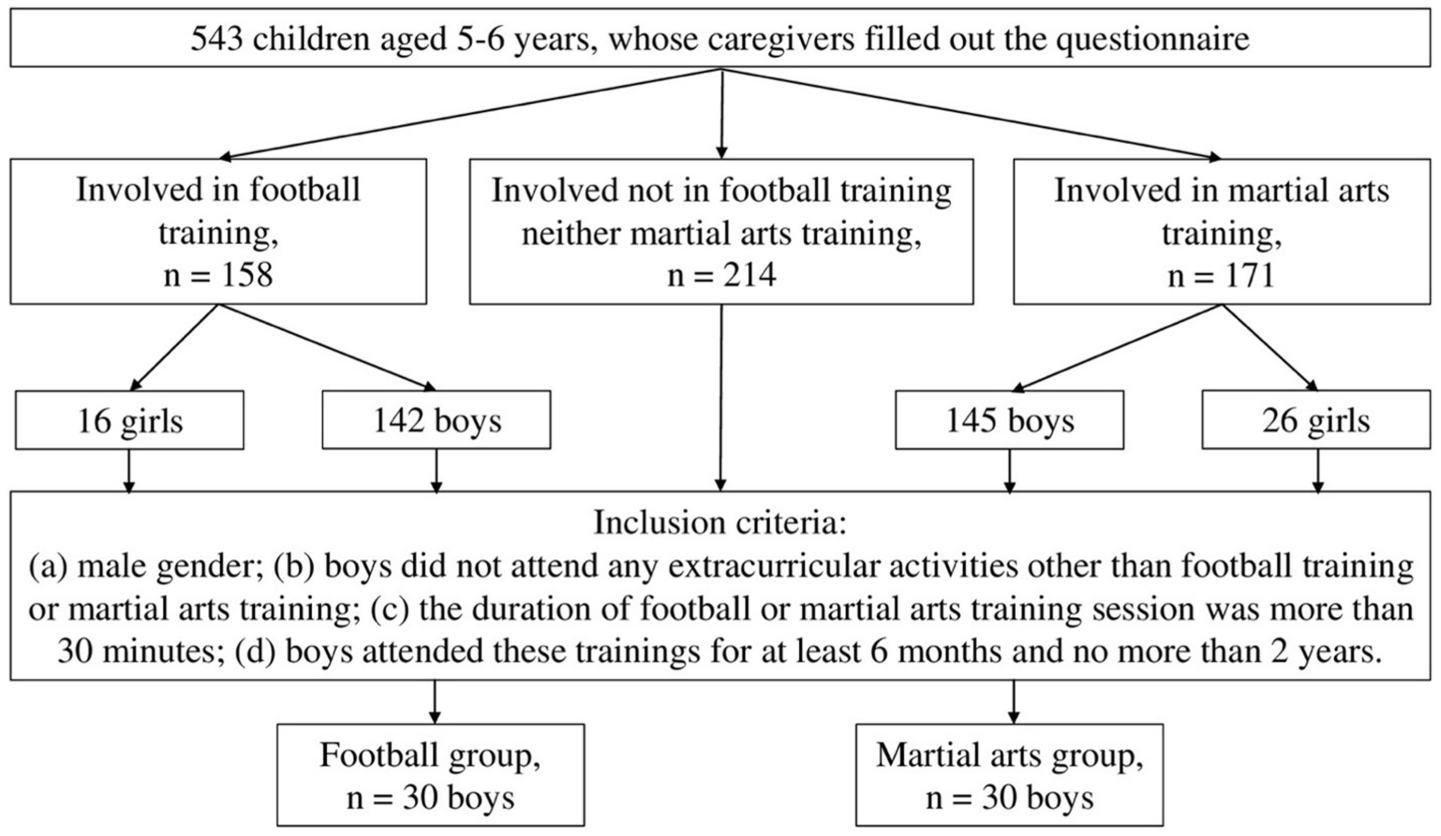
Inclusion criteria and formation of the sample.

Figure 1 illustrates the formation of the sample. Initially, caregivers of 543 typically developing children filled out the questionnaire. Of these, there were 158 children (of whom 90% were boys) who were involved in football training and 171 children (of whom 85% were boys) who were involved in martial arts training. After taking into account all the inclusion criteria described above, only 60 boys remained.

The study sample thus consisted of 60 boys aged 5–6: 30 boys involved in football training (the football group), and 30 boys involved in various forms of martial arts training (the martial arts group). In the martial arts group, there were 7 boys involved in karate, 7 in sambo, 4 in boxing, 4 in judo, 2 in capoeira, 2 in taekwondo, and 1 boy each for the sports of kickboxing, freestyle wrestling, jiu-jitsu, and mixed martial arts.

### 3.3 Measures

The following subtests, validated for the Russian population (Veraksa et al., 2020), were used to assess EF skills.

The NEPSY-II subtest ‘Sentences Repetition’ (Korkman et al., 2007) was used in the assessment of verbal working memory. In this subtest, the child has to memorize one by one sentences that increase in complexity in structure and length. There are 17 sentences in total. For each correctly reproduced sentence 2 points are given, for a sentence with either 1 or 2 mistakes – 1 point, for a sentence with more than 2 mistakes – 0 points. If a child receives 0 points for 4 consecutive sentences, this subtest is canceled. The maximum total score is 34 points.

The NEPSY-II subtest ‘Memory for Designs’ (Korkman et al., 2007) was used in the assessment of visuospatial working memory. In this subtest, the child has to remember the location and content of cards with geometric designs on a field of 16 cells. First, the child is shown the field of cards for 10 s. Then he/she has to choose the right cards from the cards offered by memory and put them on the right cells of the field. The subtest consists of four trials (4, 6, 6, and 8 cards in each trial). In this subtest 3 parameters are evaluated: the content component of visuospatial working memory (the content score, maximum score is 48), the spatial component of visuospatial work-ing memory (spatial score, maximum score is 24), and the combination of spatial and content components of visuospatial working memory, i.e., successful recall of both the location and content of the visual stimulus (bonus score, maximum score is 48). These three parameters make up the total score. The maximum total score is 120.

The NEPSY-II subtest ‘Inhibition’ (Korkman et al., 2007) was used in the assessment of inhibition. In this subtest, the child is shown stimulus sheets comprising sequences of 40 geometric shapes. The first two stimulus sheets show circles and squares, whilst the second two stimulus sheets show up and down arrows. The child is required to name the opposite of what is depicted at speed (e.g., say ‘circle’ when he/she sees a square, and vice versa). The time taken to complete these tasks and the number of errors are taken into account. These are then converted to a combined scaled score from 1 to 20 points based on the corresponding tables.

The ‘Dimensional Change Card Sort’ (Zelazo, 2006) was used to assess the child’s cognitive flexibility. In this subtest there are cards with boats and hares, the boats and hares can be blue or red in color. Some of the cards have a black border around the perimeter of the card. In the first trial, the child has to sort the 6 cards by color. In the second trial, the child has to sort the cards by the picture of a boat or a hare. In the third trial, the child has to sort the cards based on the presence or absence of the black border. For each correctly sorted card the child gets 1 point. The maximum total score is 24.

In order to collect information on sports training participation, a questionnaire for caregivers was used. One of the questions on the questionnaire was to find out whether the child attends sports training. It was necessary to specify the kind of sport, how many times a week the sports training session takes place, for how many minutes a session lasts, and how many months the child has been attending this training.

To describe the sample, the questionnaire included questions on the child’s gender, date of birth and place of residence, as well as two questions about the socio-economic characteristics of the family: the education level of the child’s mother (the number of years of education since the beginning of school), and the family income level (calibrated as below average, average, or above average).

### 3.4 Data analysis

Data analysis was performed using Jamovi version 2.0.0.0. The Shapiro–Wilk test indicated that the variables did not follow a normal distribution (only verbal working memory and child’s age followed normal distribution). As the data were not normally distributed, the data are presented as median and range (min; max) for continuous variables and given as frequency (%) for categorical variables. As the data were not normally distributed, further analysis was conducted using nonparametric tests.

First, the Mann–Whitney-test (*U*) was used to assess differences in the EF skills, specifics of attendance at sports training and socio-demographic characteristics between the football group and the martial arts group. The effect size of the Mann–Whitney *U* test was based on the Rank biserial correlation value (*r*_rb_): *r*_rb_ < 0.10 represented very small effect, 0.10 < *r*_rb_ < 0.29 small effect, 0.30 < *r*_rb_ < 0.49 moderate effect, and *r*_rb_ ≥ 0.5 large effect (Cohen, 2013). Given the multiple pairwise comparisons conducted between the groups, a Bonferroni correction was applied to control for the inflated Type I error rate associated with multiple testing. The conventional significance level of *p* = 0.05 was adjusted using the Bonferroni correction. With m = 13 pairwise comparisons (7 EF skills, 3 characteristics of sports training attendance, 3 socio-demographic characteristics), the adjusted significance level for each individual test was set at *p* = 0.05/13 = 0.004.

Second, to ensure that specifics of attendance at sports training and socio-demographic characteristics dis not underlie differences between groups in EF skills these variables were controlled. Generalized linear models were performed to test sports training group (football training or martial arts), specifics of attendance at sports training (number of sessions per week, duration of one session in minutes, time the child has been attending training in months), and socio-demographic characteristics (maternal education, child’s age, family’s income) as EF skills predictors. The significance level was set at *p* = 0.05.

## 4 Results

Table 1 reports the comparison of the football group and the martial arts group by EF skills. All components of visuospatial working memory (content score, spatial score, bonus score, total score) were significantly higher in the football group than in the martial arts group. The effect size was large for the ability to be able to remember the spatial location of stimuli (*r*_rb_ = 0.51). The effect was moderate for the ability to remember content (*r*_rb_ = 0.34), the ability to remember both location and content (*r*_rb_ = 0.36), and the overall visuospatial working memory score (*r*_rb_ = 0.45). Following the Bonferroni correction, with an adjusted significance p-level of 0.004, only the comparisons between the groups by spatial component of visuospatial working memory (*p* < 0.001) and by visuospatial working memory total score (*p* = 0.003) remained statistically significant. No statistically significant differences were noted between the two groups of children on verbal working memory, inhibition, and cognitive flexibility.

**Table 1:** Comparison of football group and martial arts group by EF skills, characteristics of sports training attendance and socio-demographic characteristics.

Participant characteristics	Football group, *n* = 30		Martial arts group, *n* = 30		Differences between the groups		
	Median	Min; Max	Median	Min; Max	*U*	*p*	*r*_rb_
EF skills							
Verbal working memory	18.5	10; 25	17.5	8; 28	434	0.818	0.04
Visuospatial working memory, content score	38.5	29; 48	34.0	26; 48	297	0.023	0.34
Visuospatial working memory, spatial score	21.0	14; 24	17.5	11; 24	219	< 0.001	0.51
Visuospatial working memory, bonus score	16.0	2; 46	12.0	2; 38	286	0.015	0.36
Visuospatial working memory, total score	75.0	46; 117	62.5	46; 105	246	0.003	0.45
Inhibition	10.0	6; 17	10.0	6; 15	396	0.879	0.02
Cognitive flexibility	20.0	7; 24	19.0	9; 24	337	0.493	0.11
Specifics of attendance at sports training							
Number of sessions per week	2	1; 4	2	2; 3	406	0.791	0.04
Duration of one session, minutes	60	35; 90	60	35; 120	410	0.516	0.09
Time the child has been attending training, months	18	6; 24	12	6; 24	334	0.075	0.26
Socio-demographic characteristics							
Child’s age, months	68	60; 75	69	57; 82	373	0.349	0.14
Maternal education, years	16	13; 20	16	13; 17	413	0.908	0.02
Family income	%		%		χ^2^	*p*	*V*
Below average	10		7		0.96	0.620	0.13
Average	67		59				
Above average	23		34				

*r*_rb_ < 0.10 very small effect, 0.10 < *r*_rb_ < 0.29 small effect, 0.30 < *r*_rb_ < 0.49 moderate effect, and *r*_rb_ ≥ 0.5 large effect.

Table 1 also reports the comparison of the football group and the martial arts group on characteristics of sports training attendance and socio-demographic characteristics. In both groups, boys attended sports training on average 2 times a week; each session lasted approximately 60 min and at the time of the collection of data the average period of attendance was 18 months in the football group and 12 months in the martial arts group. The average age of children in the groups was between 68 and 69 months. Most of the boys in both groups came from families with a middle socioeconomic status. In both groups, boys had mothers with 16 years of education on average. In both groups, 90% or more of the children came from families with an average or above-average income level. There were no statistically significant differences between the two groups of children in all these variables.

Table 2 reports the series of generalized linear models with the sports training group (football training or martial arts training), the specifics of attendance at sports training, the socio-demographic characteristics as predictors, and visuospatial working memory scores as outcome variables. The only model with sports training group as a significant predictor (*p* = 0.004) was the model with spatial component of visuospatial working memory score as outcome variable (Model info: *R^2^* = 0.312, *p* = 0.010). In this model, the specifics of attendance at sports training, and the socio-demographic characteristics as predictors except for the child’s age were not significant predictors.

**Table 2:** Generalized linear models with group (football training or martial arts training), specifics of attendance at sports training, socio-demographic characteristics as predictors and each visual working memory score as outcome variable.

Independent variables	*B*	*SE*	β	*p*
Outcome variable: Visual working memory, content score; Model info: *R*^2^ = 0.129, *p* = 0.447				
Intercept	36.9518	0.7384	< 0.001	< 0.001
Group (football training or martial arts training)	−2.7024	1.580	0.067	0.094
Number of sessions per week	0.017	1.026	1.018	0.987
Duration of one session in minutes	−0.080	0.052	0.923	0.127
Time the child has been attending training in months	−0.034	0.122	0.967	0.782
Maternal education	0.203	0.580	1.226	0.728
Child’s age	−0.060	0.188	0.942	0.752
Family income	−0.282	1.384	0.754	0.839
Outcome variable: Visual working memory, spatial score; Model info: *R*^2^ = 0.312, *p* = 0.010				
Intercept	19.045	0.460	< 0.001	< 0.001
Group (football training or martial arts training)	−2.985	0.985	0.051	0.004
Number of sessions per week	0.323	0.323	0.639	0.616
Duration of one session in minutes	−0.023	−0.023	0.032	0.471
Time the child has been attending training in months	0.002	0.002	0.076	0.984
Maternal education	0.409	0.362	1.505	0.264
Child’s age	−0.270	0.117	0.764	0.026
Family income	−0.508	0.862	0.602	0.558
Outcome variable: Visual working memory, bonus score; Model info: *R*^2^ = 0.177, *p* = 0.210				
Intercept	15.842	1.360	< 0.001	< 0.001
Group (football training or martial arts training)	−6.668	1.890	0.001	0.026
Number of sessions per week	−2.540	2.910	0.079	0.185
Duration of one session in minutes	−0.005	0.095	0.995	0.959
Time the child has been attending training in months	−0.021	0.225	0.979	0.927
Maternal education	−0.435	1.069	0.647	0.686
Child’s age	−0.287	0.346	0.751	0.411
Family income	2.581	2.549	13.213	0.316
Outcome variable: Visual working memory, total score; Model info: *R*^2^ = 0.197, *p* = 0.146				
Intercept	71.840	2.245	< 0.001	< 0.001
Group (football training or martial arts training)	−12.355	4.805	< 0.001	0.013
Number of sessions per week	−2.200	3.119	0.111	0.484
Duration of one session in minutes	−0.108	0.157	0.898	0.493
Time the child has been attending training in months	−0.053	0.372	0.948	0.886
Maternal education	0.177	1.764	1.193	0.921
Child’s age	−0.617	0.572	0.540	0.286
Family income	1.791	4.207	5.996	0.672

## 5 Discussion

This pilot study addressed the following research question: how do EF skills differ between 5 and 6-year-old boys attending football training and boys attending martial arts training? It was revealed that 5–6-year-old boys who took football training for at least 6 months had higher spatial component of visuospatial working memory and visuospatial working memory total score than their peers who took martial arts training for at least 6 months. Moreover, generalized linear model only for spatial component of visuospatial working memory as outcome variable was significant. In this generalized linear model, the specifics of attendance at sports training, and the socio-demographic characteristics except for child’s age were not significant predictors of spatial component of visuospatial working memory. Therefore, the differences between the groups will be considered only by this variable.

Boys involved in football training have been shown to have higher scores in spatial components of visuospatial working memory compared to those involved in martial arts training. This difference can be explained by the fact that in football a child has to take into account many visual stimuli (e.g., ball movement, field markings, movements and actions of team members and opponents) from the beginning of football education, which actively engages the visuospatial working memory. These findings are consistent with the results of a meta-analysis by Wu et al. (2025). The meta-analyses of 21 studies showed that children who played football and basketball had higher visuospatial working memory scores than those children who did not play sports (Wu et al., 2025). During football sessions children need to follow the position and movement of the ball in space, that is, react quickly to spatial information and operate it in visuospatial working memory. Thus, football training fosters spatial component of visuospatial working memory.

The higher level of spatial components of visuospatial working memory in boys from the football group may also be related to the different specifics of the process of teaching football and martial arts. From the beginning of learning football, young children learn to handle the ball and have to react to unexpected trajectories of the ball’s flight. From the first sessions, children practice dribbling around obstacles, passing the ball in pairs, and playing football in groups of 3 or 4 children (Bangsbo, 2000; Ivanov and Kuznetsov, 2021). At the beginning of martial arts learning, children are more likely to practice fighting techniques on their own and engage in general physical training (Stamenković et al., 2022). In preschoolers, martial arts training is non-contact without punches (Borisova and Gubzhokov, 2013). Children do exercises one by one and train exercises during classes (Gotay, 2013). That is to say, when learning football, the educational process from the beginning corresponds to an open-skilled sport. In the case of martial arts training, at the preliminary stage of the educational process, the activities correspond to a closed-skilled sport. Thus, it can be assumed that football training for 5–6-year-old boys may be more favorable in supporting the development of EF skills than martial arts training. On the basis of the pilot study, we suppose that in 5–6-year-old boys, football training, compared to martial arts training, may have greater potential for the development of visuospatial working memory. It is important to note that, as this is not a longitudinal study, the findings may be explained by baseline group differences in the development of EF skills. Perhaps the boys who chose to participate in football did so because they already possess superior visuospatial working memory skills.

Martial arts training may also have potential for the development of visuospatial working memory. In martial arts, athletes have to process a substantial degree of visual information simultaneously, such as the opponent’s position, the position of the body parts, the color of the floor, and the color of equipment (Romanenko et al., 2025). Thus, martial arts training may positively influence the development of visuospatial abilities. Several studies have reported higher visuospatial abilities, specifically object perception, in children involved in martial arts training compared to sedentary children (Alesi et al., 2014). However, this effect has been reported in those who have trained in martial arts for 3 years or more (Alesi et al., 2014; Muiños and Ballesteros, 2013). We assume that the effect of martial arts training on a child’s visuospatial abilities may be observed after a longer training period and may be predominantly for visuospatial perception but not for visuospatial working memory.

In this pilot study, there were no differences between groups in EF skills other than visuospatial working memory. We assume that at 5–6 years of age, sports training, (which boys attended on average twice a week), did not have a significant effect on EF skills. The development of EF skills at 5–6 years of age is more strongly influenced by other factors, such as communication with the child’s caregivers and peers, dramatic play, as well as the educational environment (Madanipour et al., 2025; Xu et al., 2024; Doebel and Lillard, 2023). We believe that both forms of training – football training and martial arts training – can equally influence the development of all EF skills except visuospatial working memory. Thus, at the initial stage of learning, in both forms of sports training, children need to listen equally to the instructions and explanations of the coach. This requires verbal working memory. Regarding the child’s inhibition, this is equally necessary for the two groups of 5–6-year-old children because they learn new movements and new rules and terms when undergoing training for football and martial arts. This requires them to follow instructions, be able to restrain inappropriate impulses and be disciplined. Cognitive flexibility in both football training and martial arts training is likely to be required to a small extent in the early stages of educational process. Because the coach gives enough time to practice each skill and the skills are learnt sequentially. The child is not required to switch between alternative strategies, frequent instructions and contexts. It can be assumed that the effect of football training and martial arts training on different EF skills becomes more pronounced at an older age, when the football or martial arts training process becomes more cognitively demanding.

## 6 Strengths and limitations

The strength of this pilot study is that the boys who were studied were not involved in any other extracurricular activities other than football or martial arts training. It is also a strength of the study that the period of sports training attendance, the duration of a session, or the number of sessions per week were not predictors of spatial component of visuospatial working memory. Nor were maternal education level, or family income level. That is, neither socioeconomic status nor the characteristics of the sports training they attended influenced the difference obtained between groups in spatial component of visuospatial working memory. In addition, a strength of the study is that the boys attended sports training for a longer period of time, more than 6 months.

Limitations of this study include the small sample size, the sample consisting of boys only, and the correlational design. To understand how football training and martial arts training affect the EF skills development, longitudinal study is needed. The information about the training type and attendance was obtained entirely from caregiver questionnaires. In our research, there is a lack of data regarding non-extracurricular sports participation (for example, playing football or practice martial casually with friends or family). Also, the limitation of this study is that the martial arts group consisted of boys practicing various forms of martial arts (karate, sambo, judo, boxing, etc.), which likely differ in structure, intensity, and cognitive demands. Grouping different forms of martial arts together reduced internal consistency and limited the validity of comparisons between groups. However, we believe that at the initial stage of the educational process, all forms of martial arts do not differ much, as they all include general physical training. Additionally, the limitation of the study is that most of the boys who participated in the study came from families with average socioeconomic status and lived in large cities.

## 7 Conclusion

This pilot study showed that 5–6-year-old boys attending football training have higher spatial component of visuospatial working memory than their martial arts peers. Although this was a pilot study, the findings suggest that football training at preschool age may better develop visuospatial working memory, especially its spatial component. Further research aimed at clarifying this assumption may be useful in practical work with children with low spatial component of visuospatial working memory. These findings are relevant for early childhood education, as visuospatial working memory is a crucial cognitive skill for school readiness, notably influencing abilities such as mathematical reasoning, reading comprehension, and the ability to follow complex instructions.

Based on this pilot study, it is possible to outline prospects for future research in this area. Firstly, an increase in sample size is required. Secondly, longitudinal study is needed. It will allow tracking changes over time, assessing the long-term impacts of the studied variables, and establishing causal relationships more effectively. Thirdly, future research should include girls to examine whether the findings are consistent across genders or if there are significant variations that need to be addressed. Fourthly, future research should explore EF skills at each martial arts training separately. Finally, to ensure broader applicability of the results, future studies should include children from different socioeconomic, cultural, and educational backgrounds.

## Data Availability

The raw data supporting the conclusions of this article will be made available by the authors, without undue reservation.
